# Metabolomics and metabolites in cancer diagnosis and treatment

**DOI:** 10.1186/s43556-025-00362-8

**Published:** 2025-11-14

**Authors:** Minyi Cai, Haiyan Liu, Chen Shao, Tingting Li, Jun Jin, Yahui Liang, Jinhu Wang, Ji Cao, Bo Yang, Qiaojun He, Xuejing Shao, Meidan Ying

**Affiliations:** 1 Department of Surgical Oncology, Children’s Hospital, Zhejiang University School of Medicine, National Clinical Research Center for Child Health, Hangzhou, 310052 China; 2 Nanhu Brain-Computer Interface Institute, Hangzhou, 311100 China; 3 Institute of Pharmacology and Toxicology, Zhejiang Province Key Laboratory of Anti-Cancer Drug Research, College of Pharmaceutical Sciences, Zhejiang University, Hangzhou, 310058 China; 4 School of Medicine, Hangzhou City University, Hangzhou, Zhejiang 310015 China; 5 Cancer Center, Zhejiang University, Hangzhou, 310058 China

**Keywords:** Cancer, Metabolites, Metabolomics, Biomarkers, Therapeutic targets

## Abstract

Cancer is a leading cause of death worldwide. Metabolic reprogramming in cancers plays an important role in tumor initiation, malignant progression and therapeutic response. Based on this, significant progress has been made in the development of the metabolite-based early cancer detection and targeted interventions. Over the past decade, metabolomics has been widely applied to detect metabolic alterations in tumor cells as well as their microenvironment. However, an up-to-date systematic review to summarize the current metabolomic and metabolites in cancer, especially their connections to cancer diagnostics/prognostic biomarkers and therapeutic strategies, is lacking. Here, we first introduced the platforms and analytical processes of metabolomics, as well as their application in different biological matrix of tumor patients. Then, we summarized representative cancer studies in which specific metabolites was found to be act as diagnostic or prognostic/stratification biomarkers. Furthermore, we reviewed the current therapeutic strategies targeting cancer metabolism, particularly the drugs/compounds that are either market-approved or in clinical trials, and also analyzed the potential of metabolites in personalizing precision treatment. Finally, we discussed the key challenges in this field, including the technical limitations of metabolomics and the clinical limitations of therapeutic targeting cancer metabolism, and further explored the future directions such as multi-omics perspective and lifestyle interventions. Taken together, we provides a comprehensive overview from technological platforms of metabolomics to translational applications of metabolites, facilitating the discovery of novel biomarkers and targeting strategies for precision oncology.

## Introduction

Cancer is a leading cause of human death worldwide. Early diagnosis and effective treatment strategies are crucial for improving patient outcomes. The relationship between cancer and metabolism has been recognized for decades, starting with Otto Warburg’s observations, which found that tumor cells prefer glycolysis even in the presence of sufficient oxygen. With the deepening of research, various studies have demonstrated the changes in metabolic pathways in diverse cancers. Therefore, these specific metabolic characteristics are regarded as an important source of diagnostic/prognostic biomarkers and also open new avenues for cancer treatment strategies.

**Fig. 1:**
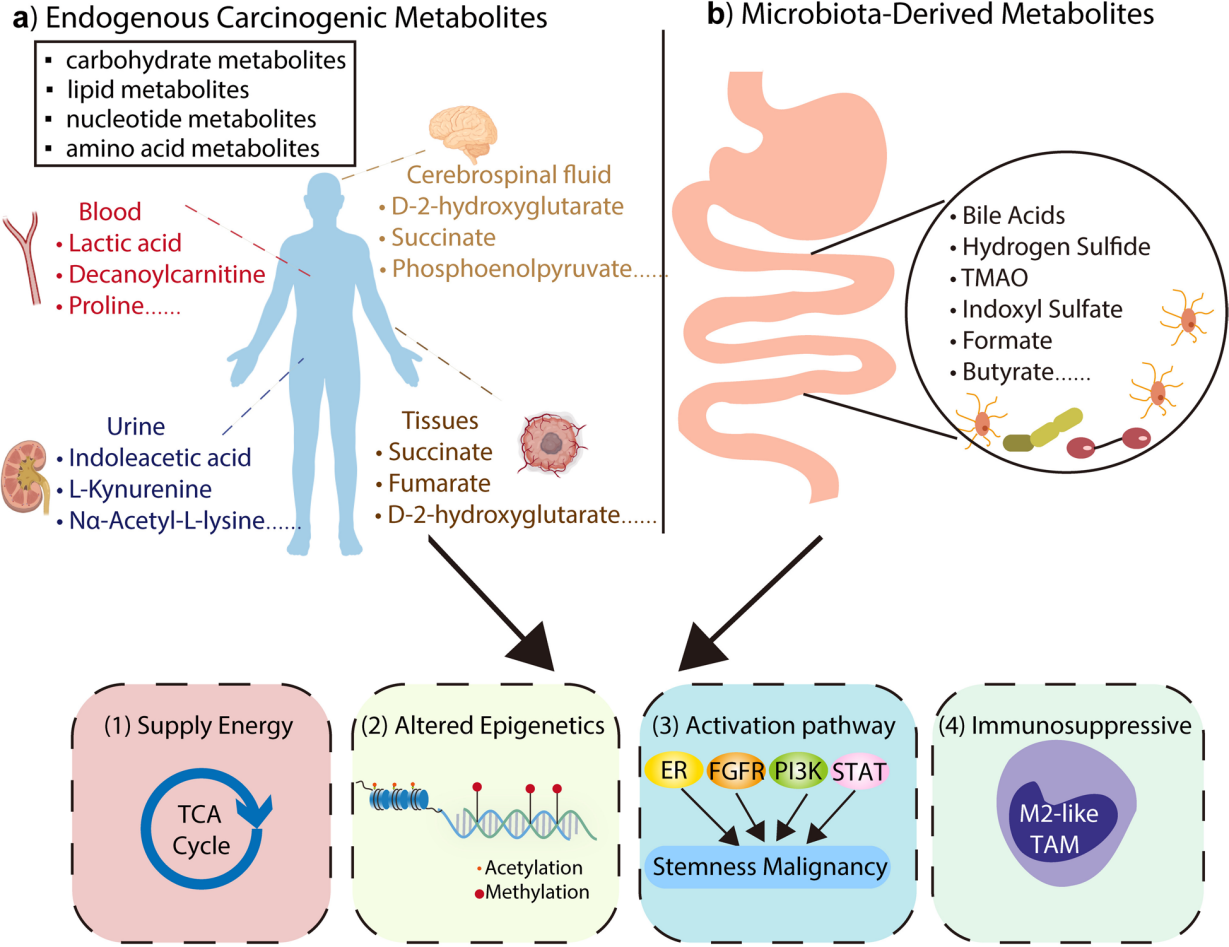
Classification and function of carcinogenic metabolites. The role of endogenous carcinogenic metabolites and gut microbiota-derived metabolites in energy supply, immunosuppressive, epigenetic alteration and pathway activation of stemness malignancy. This figure was created in BioRender. Minyi, C. (2025) https://BioRender.com/dhm2io3

Metabolites are the end products or intermediates of metabolic pathways. They have intricate connections with biological phenotypes, and their levels are influenced by both genetic and environmental factors [1]. In cancer, metabolites include endogenous metabolites produced by host cells and microbial metabolites derived from the resident microbiome (Fig. 1). According to the types of metabolites, endogenous metabolites can be classified into amino acid metabolites, carbohydrate metabolites, lipid metabolites, and nucleotide metabolites. Metabolites regulate tumorigenesis and malignant progression through multiple pathways, including supplying energy for tumor growth, participating in epigenetic modifications, activating signaling pathways and suppressing immune responses.

Metabolomics is a technique for analyzing the changes of small molecule metabolites within biological systems. In recent years, it has received significant attention in the field of cancer research as a powerful tool [2], promoting the discovery and characterization of metabolites closely related to cancer. It is worth noting that the combination of metabolomics platforms and robust computing capabilities now enables comprehensive, quantitative and spatially resolved analysis of tumor metabolic characteristics. These advances contribute to elucidating how metabolites drive tumorigenesis and progression, promoting the discovery of targeted metabolic anticancer drugs, and guide personalized precision therapies. However, there is currently a lack of a comprehensive and up-to-date review synthesizing metabolomics, metabolites, and tumor diagnosis and treatment. Therefore, this review aims to collectively summarize the current research status of metabolomics and metabolites in the field of oncology, the potential of metabolites as diagnostic and prognostic biomarkers, and the progress in clinical development of anticancer drugs targeting metabolic pathways.

In this review, we first introduce the technological platforms underlying metabolomics in cancer research, including nuclear magnetic resonance (NMR), liquid chromatography/gas chromatography/capillary electrophoresis mass spectrometry (LC/GC/CE-MS), mass spectrometry imaging, vibrational spectroscopy, and emerging single-cell and spatial imaging techniques, and discuss the advantages and limitations of each technology. We then illustrate the computational workflow of metabolomics, encompassing preprocessing, normalization and scaling, unsupervised and supervised modeling, biomarker discovery and validation, as well as pathway and network interpretation. Subsequently, we summarize research advances on metabolites serving as diagnostic and prognostic markers for various malignancies, as well as establish connections between recurrent genetic and microenvironmental drivers and key metabolic reprogramming events. Furthermore, we summarized the potential drug targets for regulating tumor metabolism and corresponding small-molecule drug development from the perspectives of carbohydrates, amino acids, nucleotides, lipids, and microbial metabolism. We also elucidated how these drugs synergize with chemotherapy and immunotherapy, and analyzed the potential applications of metabolites in personalized treatment. Finally, we explored the challenges and future directions in metabolic-based tumor diagnosis and treatment.

## Metabolomics technologies and methodologies

**Fig. 2:**
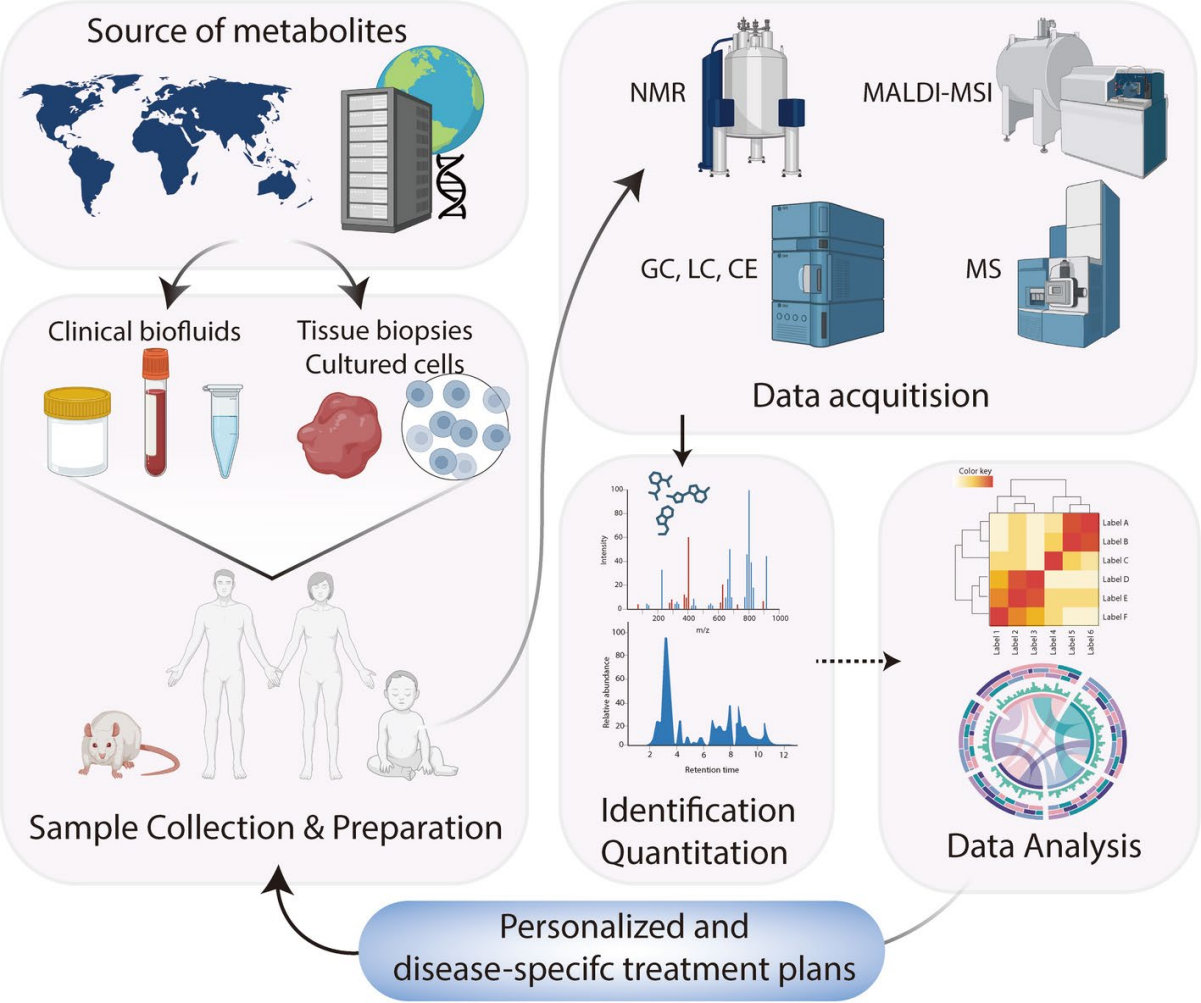
Overview of the metabolomics workflow. Metabolites are sourced from diverse populations and biological origins, including clinical biofluids, tissue biopsies, and cultured cells. Samples are processed and prepared for analysis, followed by data acquisition using techniques such as nuclear magnetic resonance (NMR), mass spectrometry imaging (MALDI-MSI), and mass spectrometry (MS), with optional separation by gas chromatography (GC), liquid chromatography (LC), or capillary electrophoresis (CE). The acquired spectra are subjected to identification and quantitative analysis, and the resulting metabolite profiles are processed and interpreted. The integrated outcomes support data-driven personalized and disease-specific treatment plans. This figure was created in BioRender. Minyi, C. (2025) https://BioRender.com/ppaptsh

Metabolomics enables the quantification of low molecular weight metabolites associated with different pathological states by providing a comprehensive metabolic profile. The study of metabolic signatures can help discover new diagnostic and prognostic biomarkers and identify new therapeutic targets. In order to more accurately describe the metabolic changes in cancer patients, there have been many metabolomics studies; the details are shown in Table 1. In addition, the upgrading of metabolomics platforms and advanced analysis strategies have provided great advances in cancer research (Fig. 2). This section provides an overview of the major analytical platforms used in cancer metabolomics, presenting recent technological developments, as well as their advantages, limitations, and applications.

**Table 1:** Representative cancer-related metabolomics based on different platform

Platform	Cancer	Sample size	Biological matrix	Reference
HR-MAS NMR	Prostate Cancer	351	Urine	[3]
NMR, LC-MS, GC-MS	Esophageal Squamous Cell Carcinoma	560	Tissue	[4]
NMR, UPLC-MS	Bladder Cancer	18	Serum	[5]
NMR, LC-MS	Acute myeloid leukemia	119	Urine	[6]
NMR	Prostate Cancer	655	Urine	[7]
NMR	Hematologic malignancies	86	Serum	[8]
NMR	Biliary tract cancer	38	Bile	[9]
NMR	Non-Small Cell Lung Cancer	74	Plasma	[10]
NMR	Lung Adenocarcinoma	18	Plasma	[11]
NMR, LC-MS, GC-MS	Breast Cancer	253	Tissues	[12]
LC-MS	Acute Myeloid Leukemia	33	Tissues	[13]
LC-MS	Triple-Negative Breast Cancer	51	Serum, Tissues	[14]
LC-MS	Multiple Myeloma	1486	plasma, Serum	[15]
LC-MS	Ovarian cancer	50	Plasma	[16]
LC-MS	Hepatocellular carcinoma	108	Plasma	[17]
LC-MS	Acute myeloid leukemia	100	Serum	[18]
LC-MS	Acute Lymphoblastic Leukemia	211	Serum	[19]
LC-MS	Esophageal squamous cell cancer	60	Tissues	[20]
LC-MS	Triple-Negative Breast Cancer	330	Tissues	[21]
HRMS	Neuroblastoma	172	Plasma	[22]
UPLC-MS	Breast cancer	66	Urine	[23]
UPLC-MS	Breast cancer	216	Plasma	[24]
UPLC-MS	Triple-Negative Breast Cancer	88	Plasma	[25]
UPLC-MS	Cervical cancer	285	Plasma	[26]
UPLC-MS	Oral squamous cell carcinoma	72	Plasma	[27]
UPLC-MS	Papillary thyroid cancer	148	Plasma	[28]
UPLC-MS	Colorectal Cancer	30	Serum	[29]
UPLC-MS	Salivary gland tumors	30	Serum	[30]
UPLC-MS, HPLC-MS	Colorectal Cancer	197	Tissues	[31]
16S rRNA gene sequencing, LC-MS	Lung Cancer	30	Serum	[32]
16S rRNA gene sequencing, LC-MS	Gastric cancer	37	Tissues	[33]
LC-MS, GC-MS	Hepatocellular Carcinoma	50	Plasma, tissues	[34]
16S rRNA gene sequencing, GC-MS	Colorectal Cancer	80	Fecal	[35]
GC-MS	Lung Cancer	144	Urine	[36]
LC-MS, GC-MS	Prostate cancer	110	Plasma	[37]
GC-MS	Glioma	30	Cerebrospinal fluid	[38]
GC-MS, CE-MS, LC-MS	Neuroendocrine tumors	77	Plasma, tissues	[39]
CE-MS	Papillary thyroid cancer	102	Tissues	[40]
CE-MS	Oral squamous cell carcinoma	22	Saliva	[41]
MALDI-MSI	Hepatocellular Carcinoma	16	Tissues	[42]
MALDI-MSI, UPLC-MS	Gastric Cancer	1212	Plasma	[43]
MALDI-MSI, HPLC-MS	Non-Small Cell Lung Cancer	1760	Tissues, plasma	[44]
MALDI-TOF MS, LC-MS	Endometrial Cancer	51	Plasma	[45]
DESI-MSI	Breast cancer, Lung cancer	6	Tissues	[46]
DESI-MSI	Oral squamous cell carcinoma	22	Tissues	[47]
DESI-MSI	Prostate cancer	444	Tissues	[48]
ATR-FTIR	Breast cancer	74	Plasma, tissue	[49]
ATR-FTIR	Digestive Tract Cancers	166	Serum	[50]
Raman	Gastric cancer	424	Ascites	[51]
Raman	Breast cancer	80	Tissues	[52]
Raman	Glioma	46	Tissue, plasma, cell lines	[53]
Raman	Prostate cancer	142	Serum, urine	[54]

### Key analytical platforms

#### Nuclear magnetic resonance (NMR) spectroscopy

NMR spectroscopy works by placing a sample in a static magnetic field, and the nucleus will absorb and re-emit electromagnetic radiation after being excited by a radiofrequency pulse at a specific frequency. Qualitative and quantitative characterization of metabolites can be achieved by the analysis of spectral parameters such as chemical shifts, peak integration, and coupling constants. The advantages of NMR include simple sample preparation, nondestructive analytical procedures, and high reproducibility of quantitative results. Therefore, many studies have applied NMR to detect differential metabolites in cancer samples [3–12].

However, its sensitivity is relatively low compared to mass spectrometry, which limits the detection of low abundance metabolites. Recent work using high-field NMR systems, 900 MHz to up to 1.2 GHz, and cryoprobes has significantly improved the signal-to-noise ratio, thereby improving the detection limit for metabolites at low concentrations [55–58]. High-resolution magic-angle spinning (HR-MAS) NMR technology supposes solid-state interactions such as dipole coupling that cause spectral line broadening by rapidly rotating the sample at a magic angle (54.74°). This technology can directly conduct high-resolution metabolite analysis on isolated tissues and the like without damaging the structural integrity of the samples, greatly expanding the application scope of nuclear magnetic resonance in tissue sample research [3, 59]. A study using HR-MAS NMR to reveal changes in glucose metabolism, oxidative stress pathways, and neurotransmitter related metabolites in mouse models of Alzheimer’s disease [60]. Another study combined NMR and mass spectrometry (MS) with machine learning to identify metabolic alterations during the progression of esophageal squamous cell carcinoma, thereby identifying potential biomarkers [4]. A targeted NMR technique named DREAMTIME can employ multi-channel spectral filters to selectively retain signals from target molecules while eliminating non-target interference, thereby overcoming the limitations of sensitivity and spectral overlap [61].

#### Mass spectrometry (MS)-based platforms

Mass spectrometry (MS) works by ionizing sample molecules and then qualitatively and quantitatively analyzing metabolites by measuring their mass-to-charge ratio (m/z) in an electric or magnetic field. In order to improve the coverage of metabolites and the specificity of analysis in complex biological samples, MS is often combined with pre-separation platforms such as liquid chromatography (LC-MS/UPLC-MS), gas chromatography (GC-MS), or capillary electrophoresis (CE-MS).

LC-MS is especially suitable for the analysis of heat-labile and non-volatile metabolites, and therefore has been widely used in lipidomics as well as in the study of various polar metabolites. It has also been applied to detect differential metabolites in many types of cancer [13–22]. When coupled with ultra-performance liquid chromatography (UPLC) and high-resolution MS (HRMS), LC-MS platforms enable high-throughput, high-precision analysis, achieves high-throughput detection. UPLC-MS has been successfully used in multiple cancer types [23–31]. In addition, with multiple reaction monitoring (MRM) scanning mode, highly sensitive quantification of target metabolites can be achieved on triple quadrupole mass spectrometry [62–64]. For instance, lipid biomarkers detected in plasma from patients with triple-negative breast cancer have shown diagnostic promise [14, 65, 66]. Similar strategies have been used in breast [23], cervical [67], and multiple myeloma metabolomics [15]. LC-MS is also common in microbiome-related cancer studies [68]; for example, combined lipidomics and multiomics approaches in colorectal cancer have clarified associations with prostaglandin metabolism [69]. When integrated with 16S rRNA gene sequencing, LC-MS can provide a deeper understanding of metabolite differences related to the microbiota [32, 33]. Recently, processes such as E-SGMN have been developed for Orbitrap Astral MS instruments, thereby expanding the detection coverage and identification accuracy of metabolites [70].

GC-MS provides high separation efficiency for volatile or derivatized compounds and offers excellent reproducibility, particularly for organic acids, sugars, and amino acids. It has been applied to gastric cancer breath testing and fecal metabolomics, aiding diagnosis and prognosis [71], showing the inhibitory effect of microbiota-derived butyrate on hepatocellular carcinoma [34], and detecting colorectal cancer biomarkers in fecal samples [35]. GC-MS has also been used to investigate metabolic dysregulation in many types of cancers [36–38, 72]. Combined LC-MS/GC-MS approaches have identified serum biomarkers for lupus nephritis [73] and, when integrated with transcriptomics, have revealed metabolic pathways regulated by p63 in cellular senescence [74].

CE-MS separates small, charged molecules, especially highly polar metabolites not amenable to reversed-phase LC-MS, and is widely used in salivary metabolomics for oral squamous cell carcinoma and in studies of many tumors [39–41, 75, 76]. Advanced methods such as AMPP-dual-CE-MS enhance detection selectivity for bile acids, revealing novel conjugates potentially regulated by the gut microbiota [77]. CE-MS is often integrated with other modalities, as demonstrated by studies that combine NMR with CE-MS to investigate inositol phosphate metabolism [78]. The combination of pneumatically assisted nanospray desorption electrospray ionization with SS-CE-MS provides spatial localization while molecular separation and identification, overcoming the limitations of either technique alone [79].

Stable-isotope labeling with MS supports fluxomics by mapping dynamic changes in metabolic pathways that underlie cancer reprogramming. ^13^C labeling can be used to track glucose flux in phosphoinositide CE-MS analysis and assist with isomer identification [78]. 13C can also be used in combination with ^34^S and ^15^N. Multidimensional tracing of sulfur-related metabolic reprogramming using different markers can provide a deeper understanding of the metabolic networks [80].

Ultra-high-resolution MS (FWHM resolution > 100,000) can effectively enhance the ability to resolve the structure of metabolites in complex samples. Rapid identification of new metabolites can be achieved even when the resolution reaches 240,000 [81]. Multidimensional LC-MS methods broaden detection and reduce ion suppression. For example, 2D-LC-MS is better than conventional LC-MS in both coverage and separation [82, 83]. Other innovative platforms that improve the stability and coverage of analysis, such as the four-in-one online analysis system [84].

#### Mass spectrometry imaging (MSI)

Mass spectrometry imaging (MSI) is a technique that enables label-free visualization of the distribution of metabolites in tissue sections. This technique can provide precise molecular-specific information while preserving the complete spatial structure. MSI lays the technical foundation for spatial metabolomics by mapping the location and relative abundance of metabolites in a histological context [85]. Spatial metabolomics combines high-resolution molecular analysis with spatially resolved tissue analysis to reveal the metabolic heterogeneity and its structural organization relationship in the tissue microenvironment.

Common MSI techniques include matrix-assisted laser desorption/ionization (MALDI-MSI), desorption electrospray ionization (DESI-MSI), secondary ion mass spectrometry (SIMS-MSI), and laser ablation electrospray ionization (LAESI-MSI). MALDI-MSI needs to cover the surface of tissue to absorb laser energy and achieve soft ionization. It can be widely used for the detection of lipids, peptides and proteins, and has been extensively applied in cancer research [42–45, 86–88]. Studies in gliomas have shown differences in lipid profiles between tumor and neuronal regions [88], and in lung cancer, MALDI-MSI has linked the distribution changes of nine lipid molecules with alterations in glycerophospholipids and glycerolipid metabolic pathways [44].

The advantage of DESI-MSI is that no matrix is required, it can be operated at normal temperature and pressure, and almost no fragment signal is generated, which is especially suitable for small molecule metabolite analysis [46, 87, 89, 90]. This approach has been applied to assess tumor margin status in oral squamous cell carcinoma [47] and to resolve positional phospholipid isomers in hepatocellular carcinoma [91]. SIMS-MSI is distinguished by its ultra-high sensitivity and spatial resolution. It can not only detect the metabolic changes of sphingolipids in the state of hyperglycemia [92], but also realize the collection of metabolic fingerprints at the single cell level by combining with single-cell metabolic phenotype analysis technology [93]. LAESI-MSI combines infrared laser ablation with electrospray ionization. It can be used to analyze the metabolism of hundreds of single cells in a tissue in situ, and the information of metabolic heterogeneity within the tissue can be completely preserved [94].

With technological advances, MSI is developing towards subcellular resolution and multimodal integration. For example, in combination with hematoxylin and eosin staining, a role for adipose triglyceride lipase in the regulation of triglyceride and choline levels in prostate cancer was confirmed [48]. The newly developed scheme of quantum cascade laser mid-infrared imaging, matrix-assisted laser desorption ionization mass spectrometry, and ion mobility prefractionation fractionation map has further elevated the speed and accuracy of metabolite spatial identification to new levels [95].

#### Vibrational spectroscopy

Infrared (IR) and Raman spectroscopy enable label-free and rapid analysis of a variety of samples by detecting molecular vibration signals. FTIR is commonly used for the analysis of cancer tissues, including lung [96], breast [97], liver [98], and endometrium [99], and is often combined with Raman spectroscopy to enable early diagnosis based on plasma spectral signatures [96, 100]. ATR-FTIR can minimize the interference of sample preparation and successfully distinguish breast cancer subtypes as well as types of digestive tract cancers [49, 50]. Raman spectroscopy has provided fine molecular maps in a variety of cancer studies [51, 52, 101–104], and combined with machine learning platforms such as APOLLO, it has further improved the accuracy of tumor classification [53, 54].

#### Single-cell metabolomics

Single-cell metabolomics (SCM) can analyze metabolites at single cell resolution and reveal the heterogeneity of tumor microenvironment. Platforms include nanoLC-MS, microfluidic ESI-MS, and single-cell MALDI-MS/SIMS, with cell isolation achieved by LCM or micromanipulation. Applications include metabolic comparisons between tumor and immune cells, tracking of cancer stem cells, and studies of drug resistance. SCENITH can detect the degree of energy metabolic pathway dependence of rare cell populations [105]. HT SpaceM has significantly improved data reproducibility by integrating MALDI-MS imaging with high-throughput workflows [106]. Although it still faces limitations in sensitivity and coverage, the combination of SCM with single-cell transcriptomics, spatial omics and machine learning is expected to achieve accurate analysis of tumor metabolic networks.

#### Multi-platform approaches

The integration of metabolomics with genomics, transcriptomics, proteomics and microbiome has effectively correlated molecular alterations with metabolic phenotypes [107, 108], improved colorectal cancer diagnosis [35], revealed metabolic driver mutations in clear cell renal cell carcinoma (ccRCC) [109], and characterized immunosuppressive neutrophil reprogramming in triple-negative breast cancer [110]. Multi-omics validation can often discover mechanisms ignored by single-omics studies. With the continuous progress of data integration technology, this strategy will provide assistance for the development of precision medicine.

### Data analysis approaches

Metabolomics data analysis converts raw MS or NMR data into interpretable biological information through a continuous workflow [111, 112] (Fig. 2). It begins with preprocessing to standardize data formats and align the signals, followed by normalization and scaling to correct technical variation. Then, unsupervised and supervised statistical analyses explore the data structure, identify discriminative features, and build predictive models. These results will be used for biomarker discovery and validation and for pathway and network analysis. The results map metabolic changes to biological systems, thereby supporting mechanistic understanding and precision medicine applications.

#### Pre-processing

The goal of preprocessing is to convert the raw spectrogram data from different instrument manufacturers into a uniform open format and to correct and organize the signal information. For NMR data, the nmrML Converter converts Bruker, JEOL, or Agilent files into a standardized format while recording acquisition parameters and metadata [113]. MS data are often converted to mzXML or mzML format by ProteoWizard, which supports data generated by major mass spectrometers such as AB Sciex, Agilent, Brooke, Thermo Fisher, and Waters [114].

After format conversion, NMR data preprocessing usually includes the steps of peak detection, phase correction, baseline correction, peak alignment and segmentation integration. The commonly used tool is NMRProcFlow [115], BATMAN [116], or Mnova [117]. MS data pre-processing typically includes peak detection, deconvolution, peak grouping, retention time alignment, and gap filling, and can be implemented with tools such as XCMS [118], Progenesis QI [119], OpenMS [120, 121], MZmine [122], and Thermo Fisher’s TraceFinder. Recently developed tools include SAND [123], MetaboLink [124], SMQVP [125], MetaboLabPy [126], mcfNMR [127], and MIRTH [128]. For instance, MIRTH uses rank transformation and non-negative matrix factorization technology to detect metabolite correlation between multiple groups of data and fill in missing values, thereby improving metabolite coverage.

#### Normalization and scaling

After the peak intensity matrix is generated, the systematic errors introduced by sample loading, instrument fluctuations and batch effects need to be corrected by normalization and standardization. Common normalization strategies include total ion current (TIC) normalization, internal-standard correction, and probabilistic quotient normalization (PQN). Data transformation methods such as log transformation or square root transformation can reduce the skewness and heteroscedasticity of the data. Pareto normalization and unit variance normalization could balance the contribution of low abundance and high abundance metabolites in the statistical model.

Widely used platforms such as MetaboAnalyst [129–131], metaX [132], IP4M [133], and NOREVA [134] incorporate these options and batch-effect correction tools, including batchCorr [135] and MetaboQC [136]. Recently proposed local neighborhood normalization (LNN) methods accurately correct for dilution effects while maintaining biological heterogeneity by constructing a local neighborhood reference spectrum for each sample [137].

#### Unsupervised statistical analysis

Unsupervised statistical analysis is used to explore the intrinsic structure of data without preset group labels. Common techniques such as principal component analysis (PCA), hierarchical cluster analysis (HCA), and self-organizing maps (SOM) can reveal sample distribution patterns, potential groups and possible outliers [138, 139].

In recent years, nonlinear dimension reduction and graph-based clustering methods have been introduced into metabolomics data exploration. Uniform Manifold Approximation and Projection (UMAP) provide low-dimensional visualizations of high-dimensional metabolic data while maintaining local and global structure [140, 141]. The Leiden algorithm can achieve stable and high-resolution community detection in the similarity network, and often cooperate with UMAP to define sample subgroups in the low-dimensional space [142]. In cancer metabolomics, these methods are used to distinguish metabolic profiles of patients from controls, identify heterogeneity within disease subtypes, and visualize batch effects across cohorts. Software platforms such as MetaboAnalyst, KIMBLE [143], and Workflow4Metabolomics [144] support PCA, UMAP, clustering, and other unsupervised analyses.

#### Supervised statistical analysis

Supervised analyses maximized discrimination between groups with the use of known group labels. Established methods include partial least squares discriminant analysis (PLS-DA), orthogonal PL-DA (OPLS-DA), sparse PLS-DA (sPLS-DA), Least Absolute Shrinkage and Selection Operator (LASSO) regression, k-nearest neighbors (KNN), and support vector machines (SVM) [145–150]. In order to reduce overfitting and ensure the generalization ability of the model, strict cross validation and permutation test are needed.

The scope of supervised analysis has expanded to include ensemble learning and deep-learning, covering random forests [151–153], gradient-boosting decision trees (GBDT), extreme gradient boosting (XGBoost) [154–156], LightGBM [157, 158], and convolutional neural networks (CNNs) [159, 160]. The emerging graph neural networks (GNNs) uses the connections between metabolites and metabolites, pathways and pathways for prediction modeling. Transformer learns the long-term dependence between multi-omics features through the attention mechanism, so as to achieve interpretable multi-marker integration in cancer classification and biomarker discovery.

Platforms such as MetaboAnalyst, metaX, IP4M, MMEASE [161], and WebSpecmine [162] have integrated many of these algorithms to support an end-to-end analysis flow from feature selection to model training and evaluation. This technological upgrade has pushed supervised metabolomics from identifying individual biomarkers toward building multi-marker predictive models for precision diagnostics and patient risk stratification.

#### Biomarker discovery and validation

Biomarker discovery and validation are based on univariate and multivariate analyses to identify metabolites that show significant between-group differences, which can be replicated in independent cohorts or experimental settings. Univariate methods include t tests, Mann–Whitney U tests, and ANOVA [163]. Multivariate feature selection may use variable importance in projection (VIP), random-forest recursive feature elimination (RF-RFE), and support vector machine recursive feature elimination (SVM-RFE) [132, 161]. The performance of the model was evaluated by receiver operating characteristic (ROC) curves and area under the curve (AUC) metrics, sensitivity and specificity measures.

A directional *p*-value merging (DPM) method has been described that integrates statistical significance and directionality across multi-omics molecular features using constrained vectors, thereby improving cross-dimensional biomarker discovery [164]. Multi-platform untargeted metabolomics combining LC-QTOF-MS, GC-QTOF-MS, and amino acid profiling has been used to examine metabolic changes associated with molecular subtypes and disease progression in breast cancer, highlighting the potential of metabolomics for early diagnosis, disease monitoring, and molecular characterization [165].

#### Pathway and network analysis

Pathway and network analyses translate metabolite lists into biological context. Databases such as KEGG, HMDB, MetaboLights, and BioCyc support enrichment analysis, pathway topology evaluation, and network visualization [166, 167]. Tools including MetaboAnalyst, MetExplore, PaintOmics, OmicsNet [168], MixOmics [169], and Cytoscape can construct metabolic networks and integrate additional omics layers. In cancer metabolomics, these analyses have revealed metabolic reprogramming phenomena, including enhanced lactate fermentation linked to hypoxic tumor microenvironments and upregulated glycerophospholipid metabolism that may contribute to cancer cell membrane remodeling [170].

The continuous improvement of analytical platforms and methodologies has made the detection of metabolic network remodeling in the process of cancer occurrence and development more sensitive, broader coverage, and more accurate quantification. NMR, MS platforms, MSI, vibrational spectroscopy, and multi-omics integration have their own unique advantages, so the choice of platform should be based on research objectives, sample types and target metabolite characteristics. Robust data standardization and rigorous statistical methodology remain critical for ensuring the reliability of results and biological interpretability. In the future, the deep integration of metabolomics with genomics, proteomics and microbiome will build a more comprehensive tumor molecular map and promote the development of accurate diagnosis and individualized treatment strategies.

## Metabolites as biomarkers in cancer diagnosis and prognosis

The National Institutes of Health (NIH) Biomarkers Definition Working Group defines biomarkers as indicators that can be measured to reflect normal biological processes, pathogenic processes, or pharmacologic responses. In the field of oncology, good biomarkers can be used to achieve early detection of diseases, which can then be used to guide treatment strategies and improve patient outcomes. Ultimately, it will reduce patient mortality and reveal new therapeutic targets. Because cancer is complex and heterogeneous, prompt diagnosis and treatment remain critical to survival. However, most of the clinical biomarkers currently in use lack sufficient sensitivity and specificity, which limits their value for early detection and treatment monitoring. Now, metabolomics has emerged as a powerful approach that can complement genotype-phenotype data. This provides direct insights into active metabolic processes and dysregulated pathways in cancer. Metabolites have great potential as diagnostic and prognostic biomarkers. Therefore, the detection of metabolomics is expected to promote precision oncology and significantly improve patient prognosis (Table 2).

**Table 2:** Small-molecule metabolites associated with cancers in recent studies based on clinical samples

Cancer Type	Cancer	Category	Biological matrix	Key Metabolites	Reference
Gastrointestinal-related tumors	Liver cancer	Diagnostic	Serum	‌L-glutamic acid^1, *^, pipecolic acid^1, †^ and alpha-fetoprotein^1^ (AFP)	[171]
			Tissue and Serum	Retinol^2, †^ and retinal^2, †^ (retinol metabolism)	[172]
		Prognostic/Stratification	Tissue and Serum	Retinol^2, †^ and retinal^2, †^ (retinol metabolism)	[172]
	Pancreatic cancer	Diagnostic	Plasma	Creatine^3, *^, inosine^2, ‡^, β-sitosterol^1, †^, sphinganine^1, †^, glycocholic acid^1, †^	[173]
			Serum	Histidinyl-lysine^2, *^, Docosahexaenoic acid^1, †^ (DHA), LysoPhosphatidylCholine^4, †^ (14:0)	[174]
		Prognostic/Stratification	Plasma	succinic acid^2, §^, gluconic acid^1, §^	[173]
			Serum	Histidinyl-lysine^2, *^	[174]
	Colorectal cancer	Diagnostic	Plasma and fecal	17-metabolite panel (FAHFA^1, †^ (22:5/22:3), ACar^1, †^ (9:1), Pseudouridine^1, *^, PhosphatidylInositol^2, †^ (20:4), stearic acid^3, †^, 3β,6α-Dihydroxy-α-innol 9-[apiosyl-(1- > 6)-glucoside]^1, §^, Ganoderiol B^1^, L-Acetylcarnitine^1, †^, Dodecanoic acid^1, †^, PhosphatidylInositol^2, †^ (18:0/22:6), Allixin^1^, Uric acid^1, ‡^, Polyporusterone F^1^, Dehydroepiandrosterone sulfate^1, †^, Sulfated Hexosylceramide^1, †^ (d29:1), Capric acid^1, †^)	[175]
			Tissue	sphingomyelin^1, †^, Ceramide^1, †^, Triglyceride lipid species signature	[176]
		Prognostic/Stratification	Plasma and fecal	19-metabolite panel (Androsterone sulfate^1, †^, L-Histidine^1, *^, Dehydroepiandrosterone (DHEA) sulfate^2, †^, 13-L-Hydroperoxylinoleic acid^1, †^, Kynurenine^1, *^, Trimethylamine N-oxide^2, ¶^ (TMAO), Lithocholic acid glycine conjugate^1, †^, Methylmalonic acid^1, ‡^)	[175]
			Tissue	Triacylglycerol profile^1, †^	[176]
	Gastric cancer	Diagnostic	Plasma	10-metabolite panel (Succinate^2, §^, Uridine^3, ‡^, Lactate^1, §^, S-AdenosylMethionine^1, ‡^, Pyroglutamate^1, ‡^, 2-Aminooctanoate^1, †^, Neopterin^2, ‡^, N-Acetyl-D-glucosamine 6-phosphate^1, §^, Serotonin^1, *^, Nicotinamide mononucleotide^1, ‡^)	[177]
			Tissue	1-Methylnicotinamide^1, §^, N-acetyl-D-glucosamine-6-phosphate^1, §^	[33]
		Prognostic/Stratification	Plasma	28-metabolite panel (Symmetric dimethylarginine^1, *^, Neopterin^2, ‡^, Thymine^1, ‡^, Glucuronate^1, §^, Hydroxyproline^1, *^, Carnitine^3, †^ (14:0), Indoleacrylate^1, ‡^, Carnitine^3, †^ (8:0), Acetylalanine^1, *^)	[177]
			Tissue	β-Alanine^4, *^, Aspartic Acid^1, ‡^, Guanosine Diphosphate^1, ‡^, Glycine^2, *^	[178]
			Serum	Deoxyribose-1-phosphate^1, ‡^, S-lactoylglutathione^1, §^	[179]
	Esophageal squamous cell carcinoma	Diagnostic	Plasma	Hypoxanthine^2, ‡^, Proline betaine^1, *^, Indoleacrylic acid^1, *^, Inosine^2, ‡^, 9-decenoylcarnitine^1, †^, tetracosahexaenoic acid^1, †^, LysoPhosphatidylethanolamine^1, †^ (20:4), LysoPhosphatidylCholine^4, †^ (20:5)	[180]
			Tissue and Serum	Purine salvage metabolites (hypoxanthine^2, ‡^, xanthine^1, ‡^)	[181]
Genitourinary-related tumors	Bladder cancer	Diagnostic	Urine	Acylcarnitines^1, †^, Phosphoenolpyruvate^1, §^, Pyruvate^1, §^, Succinate^2, §^, oxoglutarate^1, §^	[182]
			Urine	D-ribose^1, §^, D-mannose^1, §^, erythritol^1, §^, desaminotyrosine^1, ‡^	[183]
			Plasma and serum	Acetylphenylalanine^1, *^, Phosphatidylcholine^3, †^ (40:7), Phosphatidylcholine^3, †^ (40:6)	[184, 185]
		Prognostic/Stratification	Serum	Inosine^3, ‡^, AFMK^1, *^, Phosphatidylserine^1, †^ (O-18:0/0:0)	[186]
	Prostate cancer	Diagnostic	Urine	Nine-metabolite panel (propenoic acid^1, †^, dihydroxybutanoic acid^1, †^, pyrimidine^1, ‡^, creatinine^1, *^, purine^1, ‡^, glucopyranoside^1, §^, ribofuranoside^1, §^, xylonic acid^1, §^, xylopyranose^1, §^)	[187]
			Urine	Phosphatidylcholine^3, †^s (34:2, 34:1)/LysoPhosphatidylCholine^4, †^ (16:0) ratio	[188]
Breast-related tumors	Breast cancer	Diagnostic	Plasma	Tyrosine^2, *^, Alanine^4, *^, Glutamic acid^2, *^, Phenylalanine^3, *^, Palmitic acid^2, †^, linoleic^1, †^, Stearic acid^3, †^	[189]
			Urine	Dimethylheptanoylcarnitine^1, §^, succinic acid^2, §^	[189]
			Plasma	Inosine^4, ‡^, Uridine^3, ‡^, Phenylalanine^3, *^, Threonine^2, *^	[190]
		Prognostic/Stratification	Plasma	Inosine^5, ‡^, Uridine^3, ‡^ (nucleotide metabolism)	[190]
Gynecologic-related tumors	Cervical cancer	Diagnostic	Plasma	Cyclohexylamine^1, *^, Carnitine^3, †^, Val-Thr^1, *^, Sinigrin^1, §^, 5,6,7,8-tetrahydro-2-naphthoic acid^1, §^	[191]
		Prognostic/Stratification	Plasma	Trimethylamine N-oxide^2, ¶^ (TMAO)	[191]
	Ovarian cancer	Diagnostic	Serum	Methionine^2, *^, Glutamine^3, *^, Asparagine^1, *^, Glutamic acid^3, *^, glycolic acid^1, §^	[192]
			Serum	Palmitic acid^2, †^ (C16:0), stearic acid^3, †^ (C18:0)	[193]
		Prognostic/Stratification	Serum	Methionine^2, *^	[192]
Central nervous system-related tumors	Glioma cancer	Diagnostic	Plasma	15-metabolite panel (including myo-inositol^1, §^, cysteine^1, *^, glycine^2, *^, proline^2, *^, N-acetylglucosamine^1, §^, etc.)	[194]
Thoracic/pulmonary-related tumors	Lung cancer	Diagnostic	Plasma	Multi-metabolite panel (β-hydroxybutyric acid^1, †^, LysoPhosphatidylCholine^4, †^ 20:3, PC ae^1†^ C40:6, citric acid^1, §^, fumaric acid^1, §^)	[195]
			Urine	Creatine riboside^3, *^, N-acetylneuraminic acid ^2,^^§^	[196]
		Prognostic/Stratification	Urine	Creatine riboside^3,^^*^, N-acetylneuraminic acid ^2,^^§^	[196]
			Serum	N-(3-Indolylacetyl)-L-Alanine^4,^^*^	[197]
Endocrine/neuroendocrine-related tumors	Neuroblastoma	Diagnostic	Plasma	49 discriminant metabolites including arginine^1, *^, glutamine^3, *^, phenylalanine^3, *^, bile acids^1, †^, oxidized lipid^1, †^	[22]
	Papillary thyroid cancer	Diagnostic	Plasma	Sebacic acid^1, †^, L-glutamine^3,^^*^, indole-3-carboxaldehyde^1, §^	[198]
		Prognostic/Stratification	Tissue	Metabolite alterations (e.g., glycerophospholipids^1, †^, amino acids^*^) integrated with proteogenomics	[40]
Head and neck-related tumors	Salivary gland tumors	Diagnostic	Serum	Serine^1, *^, Lactic acid^1, §^	[30]
	Oral squamous cell carcinoma	Prognostic/Stratification	Plasma	7 key metabolites (acetone^1, §^, sarcosine^1, *^, formate^1, *^, alanine^4, *^ (Ala), proline^2, *^ (Pro), threonine^2, *^ (Thr) and tyrosine^2, *^ (Tyr))	[199]
Hematologic-related tumors	Leukemia	Diagnostic	Serum	2-Hydroxyglutarate (2-HG) ^1,^ ^§^	[200]
		Prognostic/Stratification	Serum	2-Hydroxyglutarate (2-HG) ^2,^ ^§^	[200]

In the table, “*” highlights represent amino acid metabolites; “†” highlights represent lipid metabolites; “‡” highlights represent nucleotide metabolites; “§” highlights represent carbohydrate metabolites; and “¶” represent gut microbiota-derived metabolites. The numeral in the upper-right corner indicates the number of times the metabolite appears in this table

### Diagnostic biomarkers

With the help of metabolomics, we have found valuable diagnostic biomarkers in various types of cancer. We achieved early detection and differential diagnosis with high accuracy. The detection of metabolites in both urine and blood showed strong discrimination power and was better than traditional protein markers. This shows the potential of non-invasive methods for clinical screening. The examples below illustrate the use of metabolites as diagnostic biomarkers across representative cancers.

#### Genitourinary-related tumors

Urine profiling has revealed altered levels of acylcarnitines, glycolytic intermediates, and tricarboxylic acid (TCA) cycle intermediates, that distinguished bladder cancer (BC) from controls with high accuracy [182]. Moreover, early-stage BC detection has benefited from urine-based signatures such as sugars and polyols (e.g., D-ribose, D-mannose, and erythritol), which provide a non-invasive tool for routine screening [183]. In addition, metabolomic analyses of plasma and serum, including lipidomic analyses, have achieved near-perfect classification performance and further enabled discrimination between BC and renal cell carcinoma [184, 185]. More detailed findings on metabolic alterations in bladder cancer are available in relevant systematic review [201].

Urine metabolomic analyses have identified candidate diagnostic biomarkers for prostate cancer (PCa). Wu et al. evaluated urinary sarcosine and concluded it lacked diagnostic value; instead, a GC-MS-based panel of nine metabolites, including propenoic acid, dihydroxybutanoic acid, pyrimidine, creatinine, and purine, distinguished PCa from controls with high accuracy (AUC = 0.94) [187]. In a related study, Li et al. reported that the urinary phosphatidylcholine/lysophosphatidylcholine (PC/LPC) ratio was significantly elevated in patients with PCa patients compared with benign prostatic hyperplasia, suggesting a non-invasive diagnostic utility [188]. A summary of existing metabolomic prostate cancer biomarkers is provided in comprehensive review [202].

#### Breast-related tumors

In breast cancer, a systematic review reported frequent alterations in amino acids (tyrosine, alanine, glutamic acid, phenylalanine) and fatty acids (palmitic, linoleic, stearic), with high diagnostic accuracy observed in blood-based studies [189]. In urine, combinations such as dimethylheptanoylcarnitine with succinic acid yielded sensitivities and specificities above 85% [189]. More recently, a four-metabolite plasma panel (inosine, uridine, phenylalanine, threonine) achieved AUCs up to 0.95 across cohorts, supporting its potential as a robust diagnostic panel [190]. Additional findings are summarized in breast cancer-focused review [203].

#### Gynecologic-related tumors

In ovarian cancer, metabolomics analysis has identified several diagnostic metabolites. For example, targeted metabolomics analysis of serum revealed that methionine, glutamine, asparagine, glutamate, and glycolic acid are potential biomarkers for differentiation, with an AUC value as high as 0.95 [192]. GC-MS analysis further highlighted the changes of saturated fatty acids in cancer, such as esterified palmitic acid (C16:0) and stearic acid (C18:0). Their validated AUC values ranged from 0.70 to 0.75 [193]. A review systematically introduced the persistent changes in amino acid and lipid metabolism during cancer progression, which also supports the diagnostic role of metabolomics in early detection [204]. For a more comprehensive summary, refer to the ovarian cancer review [205].

#### Gastrointestinal-related tumors

In gastric cancer, Chen et al. developed a plasma-based, 10-metabolite diagnostic model (succinate, uridine, lactate, S-adenosylmethionine (SAM), nicotinamide mononucleotide (NMN), among others) using machine-learning methods. The model achieved high sensitivity and specificity and was proposed as a diagnostic biomarker panel [177]. In parallel, Dai et al. identified 1-methylnicotinamide and N-acetyl-D-glucosamine-6-phosphate as a tissue-based combination that distinguished gastric cancer from noncancerous tissue (AUC = 0.976), highlighting its diagnostic utility [33]. More detailed results can be found in gastric cancer-focused reviews [206].

Similarly, in hepatocellular carcinoma (HCC), a serum-based metabolite panel was identified in metabolic syndrome-positive cases and was proposed as a diagnostic tool with improved accuracy compared with AFP [171]. In addition, tissue and serum profiling identified retinol and retinal as discriminative biomarkers that distinguish HCC from cirrhosis [172]. Further details are available in HCC-focused review [207]. Moreover, diagnostic biomarkers have also been detected across many other cancers through metabolomic analyses [175, 180].

#### Thoracic/pulmonary-related tumors

In lung cancer, a plasma metabolite panel demonstrated high performance in detecting early-stage non-small cell lung cancer (NSCLC), providing superior sensitivity and specificity compared with conventional markers [195]. Furthermore, urinary metabolomic profiling identified creatine riboside and N-acetylneuraminic acid (NANA) as robust diagnostic markers, which are elevated in both tumor tissue and urine, and can detect stage I-II disease [196]. Additional results are summarized in NSCLC-focused review [208].

Beyond these, metabolomic studies have also identified potential biomarkers in other cancers such as glioma, neuroblastoma, and papillary thyroid cancer, further emphasizing the broad diagnostic potential of metabolomics [194, 198].

### Prognostic and stratification biomarkers

In addition to early detection, metabolomics can also be used for patient stratification and prognosis prediction. Changes in specific metabolites and multi-metabolite panels in cancer are significantly associated with tumor grade, recurrence risk, treatment response, and survival, thus playing an important role in developing personalized treatment strategies. In the following sections, we will focus on the application of individual metabolites and metabolite panels in prognostic assessment and risk stratification for various malignancies.

#### Genitourinary-related tumors

Studies have shown that urinary metabolite profiles can distinguish non-muscle-invasive bladder cancer from muscle-invasive bladder cancer, and changes in metabolite profiles are associated with survival outcomes [182]. Similarly, serum-based metabolite combinations also improved patient classification; for example, a signature composed of three metabolites (inosine, AFMK, and PS(O-18:0/0:0)) could accurately differentiate low-grade and high-grade tumors (AUC > 0.95) [186].

In prostate cancer, metabolomics studies have also identified changes in related metabolites that play an important role in patient prognosis and disease monitoring [187, 188]. A comprehensive review found that changes in relevant metabolites, particularly those involving amino acids, lipids, and organic acids, can help predict disease progression, recurrence, and response to treatment, and support patient stratification [209].

#### Gynecologic-related tumors

In ovarian cancer, evidence remains limited, but decreased serum methionine levels and altered lipid signatures have been reported to be associated with advanced stage and poor differentiation, suggesting potential prognostic applications [192]. In addition, biomarkers in other gynecologic-related tumors have also been analyzed and identified through metabolomic studies [191].

#### Breast-related tumors

Certain metabolites have also shown strong prognostic relevance. Altered nucleotide metabolism, characterized by elevated inosine and uridine levels, has been associated with regulatory T-cell activation in triple-negative breast cancer and predicts response to neoadjuvant chemotherapy, supporting the role of these metabolites as stratification biomarkers [190].

#### Gastrointestinal-related tumors

In colorectal cancer (CRC), Ecker et al. identified triacylglycerol species that were associated with disease-free survival and lymphovascular invasion and explicitly described them as prognostic lipid biomarkers [176]. Similarly, in gastric cancer, Kaji et al. found that β-alanine was an independent prognostic biomarker for peritoneal recurrence and overall survival [178]. Also in gastric cancer, Chen et al. proposed a plasma-based 28-metabolite prognostic panel, that significantly outperformed clinical parameters in risk stratification [177]. In addition, Nishiumi et al. reported that serum deoxyribose 1-phosphate and S-lactoylglutathione were predictive biomarkers of sensitivity to neoadjuvant chemotherapy in gastric cancer [179].

In hepatocellular carcinoma (HCC), decreased levels of retinol and retinal were associated with Edmondson grade and poorer survival, suggesting a prognostic role [172]. Broader alterations in glucose, lipid, and nucleotide metabolism were also associated with patient outcomes and have been proposed as stratification markers [210]. Moreover, metabolomics approaches have been applied to pancreatic cancer and esophageal squamous cell carcinoma to discover prognostic biomarkers [173, 174, 181].

#### Hematologic-related tumors

In hematological malignancies, particularly acute myeloid leukemia (AML), metabolomics has expanded our understanding of diagnostic and prognostic biomarkers. Serum 2-hydroxyglutarate (2-HG), elevated in IDH1/2-mutant AML, distinguishes mutant from wild-type cases and serves as a diagnostic marker; persistently high 2-HG further predicts poorer survival, underscoring its role in risk stratification [200]. Beyond 2-HG, metabolomic profiling of patients with AML has revealed signatures associated with prognosis and treatment response, suggesting their utility in patient classification [211]. Moreover, integrative genome-metabolic analysis has defined a subgroup with mutations in NPM1 and adhesives. The characteristic of subgroups is that they have different metabolic dependencies. Link genotypes with metabolic phenotypes and support patient stratification [6]. Furthermore, studies on hematopoietic stem and progenitor cells have shown that metabolic characteristics are influenced by differentiation and aging. These findings indicate metabolic changes during disease progression and are helpful for identifying potential biomarkers [212].

#### Thoracic/pulmonary-related tumors

In lung cancer, such as in stage I-II non-small cell lung cancer (NSCLC), a poor prognosis of creatine riboside in urine is related to NANA levels, [196]. In addition, in patients with advanced NSCLC receiving PD-1 inhibitors in combination with chemotherapy, progression-free survival was independently associated with a lower serum level of N- (3-indoleacetyl) -L-alanine. Demonstrating that metabolomics can be used to predict response to immunotherapy and to strati categorize affected patients [197].

Beyond these examples, many other cancer types also exhibit potentially prognostic metabolic biomarkers, such as oral squamous cell carcinoma (OSCC) [199].

Overall, identified metabolomic biomarkers for prognosis and patient stratification. It has shown clinical value in linking metabolic alterations to clinical outcomes. Therefore, the role of metabolomics in predicting survival, recurrence, and treatment response suggests its great value in precision oncology. However, larger scale studies are still needed to realize the clinical translation of metabolomics research results.

### Mechanistic insights from metabolic pathways

**Fig. 3:**
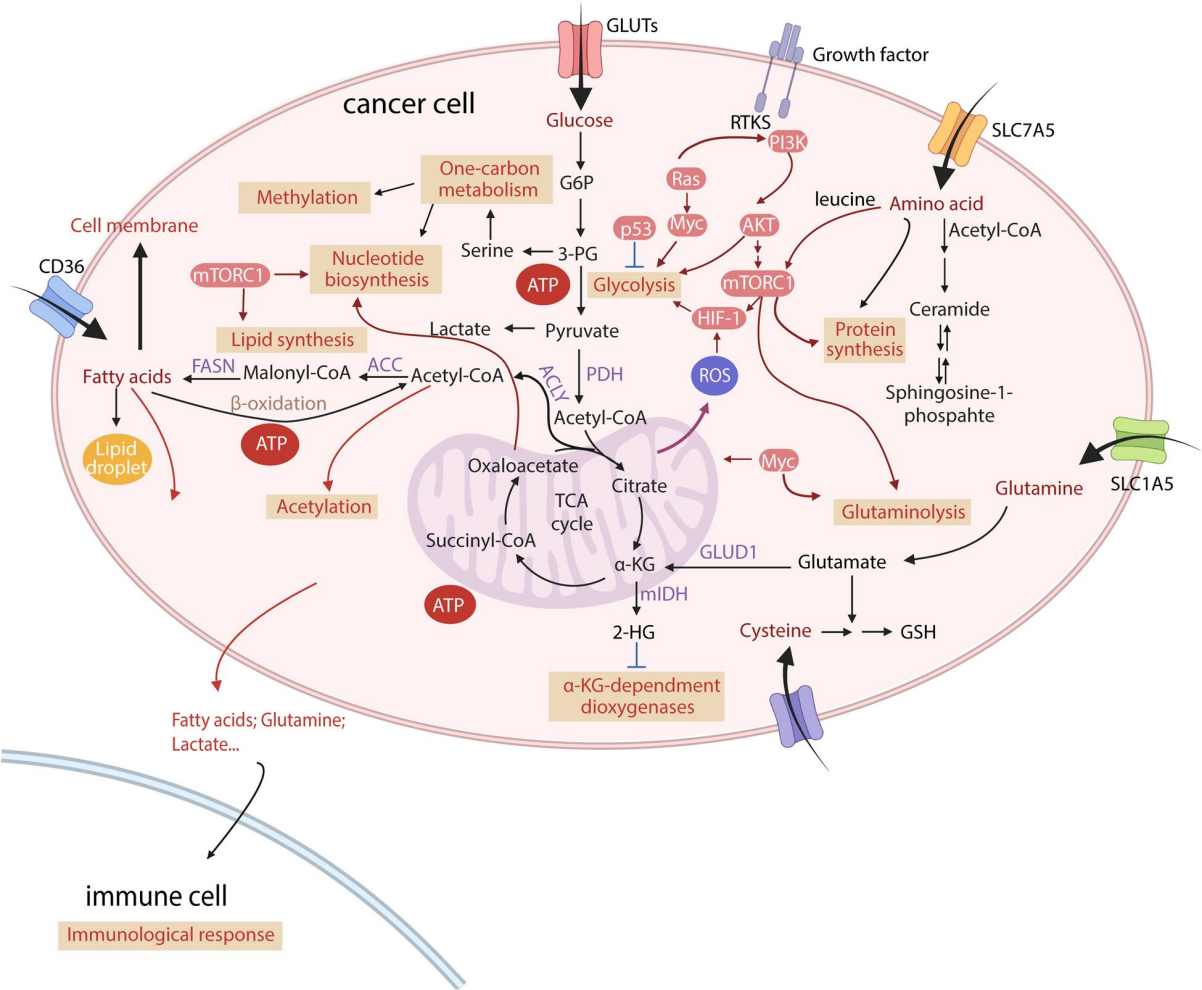
Metabolic reprogramming in cancer cells. Tumor cells undergo profound metabolic rewiring to support uncontrolled proliferation, survival, and adaptation to environmental stress. Glucose uptake through GLUT transporters is increased, fueling aerobic glycolysis (the Warburg effect) and generating intermediates for biosynthetic pathways, including serine-driven one-carbon metabolism and nucleotide synthesis. Oncogenic signaling via PI3K/AKT/mTOR and transcription factors such as MYC and HIF-1 enhances glycolytic flux, while loss of p53 relieves metabolic checkpoints. Glutamine, taken up through SLC1A5, feeds glutaminolysis, replenishing the TCA cycle and providing precursors for lipid and nucleotide biosynthesis. Mutations in IDH enzymes generate the oncometabolite 2-hydroxyglut. This figure was created in BioRender. Minyi, C. (2025) https://BioRender.com/zy0fmbc

Understanding the interplay between genetic alterations and metabolic reprogramming is central to the study of cancer-related biology. Loss of oncogenic signaling pathways and tumor suppressor genes drives tumor reorganization metabolic networks. This can be used to meet the needs of cancer cell proliferation and to adapt to the stress response of the microenvironment. In turn, metabolites produced by tumor cells alter the epigenetic landscape and cellular state. Thus, metabolomics provides a unique perspective by linking molecular drivers to functional metabolites. This helps us understand how tumor alterations lead to unique metabolic profiles and potential therapeutic weaknesses. This section summarizes the evidence linking tumor alterations and metabolic phenotypes by genetic, chemical, and microenvironmental factors (Fig. 3).

#### Glycolysis and the warburg effect

One of the most prominent and consistent features of cancer is enhanced aerobic glycolysis, which is often referred to as the Warburg effect. In this case, tumor cells first use glycolysis even under oxygen-enriched conditions. This allows rapid ATP generation and provides intermediates for biosynthesis [213]. Activation of oncogenic pathways like PI3K/AKT/mTOR and the effects of transcription factors such as HIF-1 and MYC on metabolic changes are actively driven by genetic and signaling changes rather than passive consequences [214, 215]. Conversely, the loss of some tumor suppressor genes further enhances glycolysis, such as P53. These tumor suppressor genes normally regulate glycolysis by controlling oxidative stress and metabolic checkpoints [215, 216]. This metabolic reprogramming has important implications for the immune response. This metabolic reprogramming has important implications for the immune response. The accumulation of lactic acid causes the tumor microenvironment (TME) to become acidic [217]. By down-regulating the activated receptors NKG2D and NKp46, it ultimately inhibits T cell function and NK cell activity [218, 219]. Lactic acid can also promote M2-like polarization of tumor-associated macrophages (TAMs) by regulating the ERK/STAT3 signaling pathway, and induce myeloid-derived suppressor cells (MDSCs) through the regulation of HIF-1α [219]. Taken together, these changes suggest a direct regulatory relationship between genetic drivers of cancer and metabolic reprogramming of the tumor microenvironment.

#### Amino acid metabolism

Glutamine and other amino acids are important fuels for tumor growth. The decomposition of glutamine provides substrates for the tricarboxylic acid cycle. It also supports the biosynthesis of nucleotides and lipids [216]. The MYC oncogene promotes the uptake and utilization of glutamine. However, the oncogenic metabolite 2-HG produced by IDH mutations can alter epigenetic regulation, which can drive tumorigenesis [213, 220]. In addition, leucine can activate the mTOR signaling pathway. This directly links amino acids to protein synthesis and tumor progression [221]. The metabolism of amino acids such as glutamine, tryptophan and arginine not only maintains the autonomy of cancer cells, but also plays an important role in the regulation of immune responses [222]. The consumption of glutamine by cancer cells can inhibit the cytotoxicity of CD8 ^+^ T cells by regulating IL-23. Arginase (ARG1/2) expressed by tumors and myeloid cells can degrade arginine. This will impair T cell function and promote the aggregation of regulatory T cells [219, 222]. These findings suggest that amino acid metabolism is a key node affected by genetic alterations. And amino acid metabolism significantly affects the anti-tumor immune response.

#### Lipid and fatty acid metabolism

Lipids are not only structural components but also play important roles in signal transduction. Tumor cells typically maintain de novo lipid synthesis and membrane biogenesis by upregulating the expression of various metabolic enzymes, such as fatty acid synthase (FASN), acetyl-coa carboxylase (ACC), and ATP-citrate lyase (ACLY) [213, 223, 224]. Activation of RAS and PI3K/AKT pathways would promote lipid biosynthesis, and deletion of tumor suppressor genes would enhance lipid utilization by tumor cells [213, 225]. Meanwhile, tumor cells can self-regulate fatty acid oxidation in response to changes in the tumor microenvironment. In this way, the demands of cancer cells for energy or biosynthesis are met [213, 226]. Sphingolipid metabolism plays an important role in regulating apoptosis and survival, such as the balance between ceramides and sphingolipin-1-phosphate [213]. Importantly, the lipid metabolism changes driven by oncogenes significantly affect the tumor immune microenvironment [219, 227]. Cancer cells enhance de novo lipid synthesis by fatty acid synthase (FASN), supporting their growth and damaging the function of dendritic cells [219]. In addition, lipid peroxidation is involved in other immune processes. In tumor cells, CD8 T cells take up oxidized lipids through CD36, thereby inducing lipid peroxidation. This impairs T-cell function and antitumor immunity [219]. These findings reveal processes related to lipid metabolism regulating oncogenic signals and promoting the development of tumor heterogeneity.

These findings suggest that tumor-specific genetic alterations directly affect metabolic pathways. It leads to changes in glycolysis, amino acid metabolism and lipid biosynthesis. When formulating treatment strategies targeting metabolic vulnerability, this close connection between tumor genetics and metabolism, tumor heterogeneity and plasticity is of great significance.

## Therapeutic targeting of cancer metabolism

Besides cancer biomarkers, metabolites and metabolic enzymes also represent promising therapeutic targets. Establishing metabolism-based strategies is an important approach in oncology. Metabolomics is generally divided into fields such as carbohydrate metabolism, amino acid metabolism, nucleotide metabolism, lipid metabolism, and microbial metabolism. Then, we reviewed the metabolic dysregulation of these metabolic categories and their potential as therapeutic intervention targets. Meanwhile we provided the latest progress in current drug research. The detailed information showed in Table 3.

**Table 3:** Agents targeting metabolites that are approved or in clinical trials for cancer

Classification	Metabolites	Regulation of metabolites by upstream	Tumor-promoting mechanisms of Metabolites	Agent	Phase
Carbohydrate metabolism	2-HG	IDH mutations confer neomorphic activity in the mutant protein, resulting in the conversion of αKG to 2-HG [228].	The accumulation of 2-HG results in epigenetic dysregulation via inhibition of αKG-dependent histone and DNA demethylases, and a block in cellular differentiation [228].	IDH inhibitors (Ivosidenib, Enasidenib, Olutasidenib)	Approved
	Succinate	SDH deficiency leads to succinate accumulation [229].	Succinate activate succinate receptor (SUCNR1) signaling to activate PI3K-hypoxia-inducible factor 1α (HIF-1α) axis [229].	SUCNR1 Antagonist (NF56-EJ40)	Preclinical study
	Fumarate	The loss of FH leads to fumarate accumulation [230].	Fumarate induces extensive metabolic reprogramming and drives protein succination [230].	FH inhibitor (Fumarate hydratase-IN-1)	Preclinical study
	Lactic acid	Lactic acid accumulation by highly glycolytic tumours is a strategy for immune evasion, thereby affording the tumour a growth advantage [217].	Lactic acid regulates energy metabolism and cancer cell signaling pathways [217].	MCT1 inhibitor (AZD3965)	Phase 1 (NCT01791595, Completed)
Amino acid metabolism	Asparagine	Low ASNS expression renders ALL cells highly dependent on the uptake of extracellular asparagine [231].	Asparagine-mediated protein translation is necessary for the proliferation and migration of adaptive cells [231].	Pegylated Asparaginase (Oncaspar)	Approved
	Glutamine	Genetic and microenvironmental factors can ‘lock’ tumor cells into a state of glutamine addiction and dependence on GLS [232].	Glutamine serves as a carbon source for the synthesis of lipids and metabolites via the TCA cycle [232].	GLS1 inhibitor (CB-839)	Phase 2 (NCT02861300, Completed)
	Arginine	Overexpressed argininosuccinate synthetase 1 (ASS1) leads to arginine accumulation [233].	Arginine is a second messenger-like molecule that reprograms metabolism to promote tumor growth [233].	PRMT5 inhibitor (GSK-3326595)	Phase 2 (NCT04676516, Completed)
	Methionine	Methionine is obtained through the diet [234].	Methionine is a crucial role in the regulation of SAM both in altered chromatin states, depending on p53 status [235].	MAT2A inhibitor (AG-270)	Phase 1 (NCT03435250, Terminated)
	Serine	3-phosphoglycerate dehydrogenase (PHGDH) is a key enzyme that functions as the primary rate-limiting enzyme in the serine biosynthesis pathway [236].	Serine is a key contributor to the generation of one-carbon units for DNA synthesis during cellular proliferation [236].	PHGDH inhibitor (NCT-503)	Preclinical study
	Tryptophan	IDO1 determine tryptophan deprivation and producing immunosuppressive metabolites named kynurenines [237].	Kynurenine contributes to tumor-induced immunosuppression [237].	IDO1 inhibitor Epacadostat	Phase 3 (NCT03361865, Completed)
	L-arginine	Expression of the enzyme arginase 1 (ARG1) leads to depletion of L-arginine [238].	L-arginine, a nutrient required for T cell and natural killer (NK) cell proliferation, depletion of L-arginine is a defining feature of immunosuppressive myeloid cells [238].	ARG1 inhibitor (CB-1158)	Phase 1 (NCT02903914, Completed)
Nucleotide metabolism	dNTP	dNTP biosynthesis can be promoted by the inactivation of the p53 and LKB1 tumour suppressors, or by activation of MYC, RAS and AKT oncogenes [239].	dNTP pool alterations lead to genomic instability [239].	RR inhibitor (Hydroxyurea, Gemcitabine et al.)	Approved
	Adenine	The deletion of the MTAP gene blocks the adenine salvage pathway, rendering de novo synthesis the sole route for adenine nucleotide production [240].	Adenine are fundamental and necessary for tumor cell proliferation [240].	Adenylosuccinate synthetase inhibitor (L-Alanosine)	Phase 2 (NCT00062283, Completed)
	Adenine	Under hypoxic conditions in tumors, oxygen deprivation triggers the accumulation of extracellular ATP (eATP), which is then gradually degraded to adenosine [241].	Extracellular adenosine modulates immune cell infiltration and activation via P1 purinergic receptors (A1R, A2AR, A2BR, A3R) [241].	Adenosine A2A receptor Antagonist (Ciforadenant)	Phase 2 (NCT03337698, Active)
	Adenosine	CD73 is the major enzyme responsible for its extracellular production of Adenosine [242].	Adenosine has emerged as a potent immune suppressant within the TME [242].	CD73 inhibitor (AB680)	Phase 1b/2 (NCT04381832, Completed)
Lipid metabolism	Fatty acid	Upregulation of FASN accompanies endogenous lipogenesis [224].	FA involves in cell migration and invasion, angiogenesis and escape from immune surveillance [243].	FASN inhibitor (TVB-2640)	Phase 3 (NCT05118776, Active)
	cholesterol	Upregulation of SREBPs, HMGCR, FDFT1, etc. promoted the synthesis of cholesterol [244].	Cholesterol inhibits immune-effector cells and antigen presentation [244].	HMGCR inhibitor (statins)	Phase 4 (NCT04776889, Completed)
Microbial metabolism	Secondary bile acids, LPS, TMAO, KYNA, et al	A high-fat diet improves hepatic bile acid synthesis and secretion into the gut [245].	Bile acids suppress CD8^+^ T cell effector functions [246].	Fecal microbiota transplantation	Approved

Data source: ClinicalTrials.gov

### Metabolic inhibitors and drug development

#### Carbohydrate metabolism

Carbohydrate metabolism includes a series of biochemical pathways that convert carbohydrates into energy and other biomolecules. Glycolysis is one of the most important pathways, which breaks down glucose into pyruvate to produce ATP. Further, the TCA cycle oxidizes pyruvate to generate energy. And gluconeogenesis synthesizes the glucose from non-carbohydrate precursors.

In carbohydrate metabolism, several studies have identified three canonical oncometabolites: D-2-HG, succinate, and fumarate. The dysregulation of these metabolites leads to the emergence of tumor characteristics, including hypermethylation phenotypes, alterations in metabolic pathways, and dysregulation of REDOX homeostasis. The abnormal accumulation of the metabolite D-2-HG in cancers is mainly due to IDH1/IDH2 mutations, which leads to the over-production of D-2-HG [228]. These cancer patients with IDH mutations can now receive targeted therapy with clinically approved IDH inhibitors such as Ivosidenib. Because of the mutations in succinate dehydrogenase (SDH), succinate is abnormally accumulated, promoting tumor growth and metastasis by stabilizing HIF-1α and activating SUCNR1 signaling [229]. These phenomena suggest that mutant SDH, the SUCNR1 receptor, and succinate transporters are potential therapeutic targets [247]. NF56-EJ40 is a selective antagonist of SUCNR1, which can significantly inhibit SUCNR1-mediated Gq and Gi signaling [248]. In addition, we previously found that fludarabine can be used to reduce succinate, which can restore the sensitivity of SDH-deficient AML to anticancer drugs [249]. Mutations in fumarate hydrase (FH) lead to excessive accumulation of fumarate in tumors. Currently, FH inhibitors remain in preclinical development [230].

In addition to the three identified oncometabolites, there are many other metabolites with imbalanced levels in carbohydrate metabolism, such as lactate. Dysregulation of lactate levels (including excess and deficiency) is involved in processes such as metabolic reprogramming, protein lactation, immunosuppression, chemoresistance, epigenetic changes, and metastasis, which are closely linked to adverse clinical outcomes [250]. The inhibitor AZD3965 of the lactate transporter MCT1 can effectively reduce the lactate content in cancer cells, thereby altering and inhibiting the metabolism and proliferation of tumor cells.

#### Amino acid metabolism

Amino acid metabolism plays a significant role in maintaining tumor growth and progression. Amino acid metabolism provides the raw material source for protein synthesis in cells and also promotes the activation of multiple biosynthetic and signaling pathways that are crucial for malignant tumors. Amino acid level imbalances are often observed in cancer, and such imbalances tend to contribute to the proliferation, survival and immune evasion of tumor cells.

Several amino acids have been identified as key mediators in tumor metabolism. For instance, aspartate supports nucleotide synthesis and tumor growth. Pegylated asparaginase (Oncaspar) is used to treat acute lymphoblastic leukemia by reducing aspartate levels [231]. Glutamine is the most abundant circulating amino acid, which is converted into glutamate by glutaminase (GLS), thereby providing energy and a precursor for biosynthesis. GLS inhibitors have shown great efficacy in triple-negative breast cancer, AML and non-small cell lung cancer [232, 251, 252]. Serine metabolism also supports cancer progression through nucleotide synthesis and REDOX homeostasis. Targeting its key enzyme PHGDH with compounds such as NCT-503 can impair tumor growth [236]. In addition, nutritional stress in the tumor microenvironment can also cause metabolic vulnerability. For example, arginine deprivation may trigger autophagy and apoptosis in tumor cells. Based on this phenomenon, reducing arginine levels by using arginine degrading enzymes or inhibiting PRMT5 can be used for cancer treatment [233, 253]. Similarly, it has been reported that methionine restriction can inhibit tumor growth and metastasis in epigenetic dysregulated cancers [234, 235].

In addition to regulating cell growth, amino acid metabolism also affects the immune microenvironment. Arginase 1 (Arg1) inhibits t cell function by consuming arginine. In preclinical models, the inhibition by drugs such as CB-1158 can restore anti-tumor immunity [238]. Similarly, the activation of indoleamine 2, 3-dioxygenase 1 (IDO1) in tumors consumes tryptophan and generates immunosuppressive kyurine, thereby promoting the development of IDO1 inhibitors such as epacadostat [237].

#### Nucleotide metabolism

Nucleotide metabolic disorders are a hallmark of cancer, supporting the high demand for DNA replication and RNA synthesis in rapidly proliferating tumor cells. The key enzymes in nucleotide biosynthesis are frequently upregulated in cancer and are attractive therapeutic targets [254]. Ribonucleotide reductase (RR) is a rate-limiting enzyme that converts ribonucleotides into deoxyribonucleotides and is crucial for maintaining the dNTP library necessary for DNA replication and repair [239]. RR inhibitors used in clinical practice, such as hydroxyurea and gemcitabine, can reduce the availability of dNTPs, thereby hindering DNA synthesis and inducing cell death. These drugs can be used as a single therapy or in combination with other chemotherapy drugs for multiple types of cancer.

Targeted nucleotide metabolism can also regulate the tumor microenvironment. For instance, L-alanosine, an inhibitor of de novo adenine biosynthesis, exhibits selective activity in MTAP-deficient cancers—where the loss of this key metabolic gene simultaneously disrupts both de novo and salvage pathways of adenine production [240]. Furthermore, the immunosuppressive effects of extracellular adenosine, which accumulates under hypoxic stress in the tumor microenvironment, can be countered using adenosine pathway inhibitors. These include agents targeting the adenosine A2A receptor (e.g., ciforadenant) or inhibiting CD73 (e.g., AB680), the ectoenzyme responsible for extracellular adenosine generation [241, 242].

#### Lipid metabolism

Disruptions in lipid metabolic processes are particularly prominent in cancer. Tumor cells use lipid metabolic pathways to obtain energy, build cellular membranes, and generate signaling molecules that promote growth, survival, invasion, and metastasis, modulate the tumor microenvironment, and influence treatment response [255]. To inhibit fatty acid (FA) synthesis, the fatty acid synthase (FASN) inhibitor TVB-2640 is currently in Phase II clinical trials. Similarly, to inhibit cholesterol synthesis, statins are currently under investigation as potential anticancer therapies in several clinical trials [244].

#### Microbial metabolism

Metabolites produced by gut microbiota mediate the interplay between the intestinal microbial community and cancer development, primarily modulating the tumor microenvironment and key signaling cascades in tumor and immune cells [256]. Metabolites such as secondary bile acids, lipopolysaccharides (LPS), trimethylamine N-oxide (TMAO), and tryptophan-derived compounds (e.g. kynurenic acid (KYNA)) have pro-tumorigenic effects and immunosuppressive effects [246, 256–258]. Maintaining gut-microbiome homeostasis is essential for preserving health and preventing oncogenesis [245]. To restore microbiome homeostasis in patients with cancer, fecal microbiota transplantation is currently being approved.

### Combination therapies

Building on the metabolic vulnerabilities described above, antimetabolic therapies are increasingly being integrated into rational combination regimens to enhance efficacy and delay resistance. They are most commonly combined with cytotoxic chemotherapy, immunotherapy, and targeted agents.

#### Combination with chemotherapy

Some antimetabolites are themselves used as cytotoxic chemotherapeutic agents because of the breadth of their therapeutic targets; examples include Oncaspar, hydroxyurea, and gemcitabine. In practice, these agents often achieve superior efficacy when combined with other chemotherapies. For instance, Oncaspar in combination with CVAD (cyclophosphamide/vincristine/doxorubicin/dexamethasone) may be suitable for study in younger adults with previously untreated acute lymphoblastic leukemia (ALL) [259]. A clinical trial is currently evaluating gemcitabine plus cisplatin as neoadjuvant chemotherapy for patients with high-grade upper tract urothelial carcinoma [260]. Beyond combinations of conventional chemotherapies, metabolically targeted agents are also frequently combined with chemotherapy. The combination of ivosidenib—an IDH1 inhibitor—and azacitidine has been approved by the FDA for the treatment of older adults with newly diagnosed IDH1-mutated acute myeloid leukemia (AML) [261]. Moreover, synergy between AG-270 and taxanes has been demonstrated in vitro [262].

#### Combination with immunotherapy approaches

In addition to counteracting tumor-promoting metabolic reprogramming, activating antitumor immunity is a central therapeutic priority. Consequently, antimetabolic agents are often combined with immune checkpoint inhibitors or other immunomodulatory strategies. Immune checkpoint blockade targeting the PD-1/PD-L1 axis has produced substantial clinical benefit across multiple malignancies. Building on this, dual metabolic–immune targeting can overcome resistance: combining a PD-L1 antibody–drug conjugate (PD-L1–ADC) with the monocarboxylate transporter 1 (MCT1) inhibitor AZD3965 has been shown to effectively treat tumors refractory to PD-1/PD-L1 blockade [263]. Clinically, anti-PD-1 combined with anlotinib and pegaspargase (Oncaspar) has emerged as a promising treatment backbone for localized extranodal NK/T-cell lymphoma, with mild toxicity and good tolerability [264]. In preclinical models of pancreatic ductal adenocarcinoma liver metastasis, anti-PD-1 combined with gemcitabine enhanced Th1- and M1 macrophage-mediated immunity, promoted CD8 ^+^ T-cell responses, and conferred therapeutic benefit [265]. Tumor-intrinsic fatty acid synthase (FASN) functions as a metabolic checkpoint that constrains T-cell immunity and represents a tractable target to improve T-cell-based therapies [243]. Co-inhibition of FASN with two mechanistically distinct agents, orlistat and TVB-2640, combined with an anti-PD-L1 antibody robustly suppressed tumor growth in vivo, underscoring the rationale for integrating metabolic inhibition with checkpoint blockade [266].

Beyond checkpoint inhibitors, antimetabolic therapies can be paired with other immune-activating monoclonal antibodies. AZD3965 can be combined with rituximab, a component of the standard-of-care regimen R-CHOP, for diffuse large B-cell lymphoma and Burkitt lymphoma [267]. In patients with locally advanced or metastatic KRAS wild-type pancreatic cancer, nimotuzumab plus gemcitabine significantly improved overall and progression-free survival, with a favorable safety profile [268]. In a Phase II study of relapsed high-grade astrocytoma, the FASN inhibitor TVB-2640 was well tolerated and could be safely combined with bevacizumab [269]. Metabolic modulation can also enhance CAR T-cell therapy; for example, Luu et al. reported that butyrate supplementation improved the efficacy of CD8^ +^ CAR T-cells in a murine model [270].

#### Combination with targeted therapies

Despite clear clinical benefit, conventional targeted agents often provoke adaptive resistance driven by metabolic rewiring. Concurrent metabolic inhibition can blunt or reverse these adaptations and deepen responses. For example, targeting glutaminase with CB-839 (telaglenastat) and mTOR with MLN128 (sapanisertib) overcomes metabolic adaptation to mTOR inhibition in lung squamous cell carcinoma [271]. In preclinical studies, CB-839 combined with either CDK4/6 or PARP inhibitors has also produced robust antitumor activity [272]. In the subgroup of gefitinib-resistant NSCLC patients, the combination of simvastatin enhanced the efficacy of gefitinib [273].

### Personalized medicine

Tumor-related metabolites are increasingly being used to guide personalized treatment. A typical example is 2-HG. Combining 2-HG levels with the IDH mutation status can reasonably guide the use of IDH inhibitors, thereby inhibiting the accumulation of 2-HG and improving clinical outcomes [211, 274].

In addition to tumor metabolites caused by mutations, the differences in metabolites among patients can be used to stratify and customize interventions for patients. In lung cancer, the lipid metabolism score (LMS) predicts the responsiveness to anti-PD-1 treatment. In patients with a high LMS score, combined administration of MK-1775 weakened tumor lipid metabolism, enhanced anti-PD-1 efficacy, and inhibited tumor growth [275]. Gliomas with p53 deletion alone or combined with constitutively active Notch1 signaling (N1IC) display elevated mitochondrial lipid peroxidation, increased reactive oxygen species, and glutathione depletion. These patients are significantly sensitive to the pharmacological or genetic inhibition of lipid hydroperoxidase GPX4 and the induction of ferroptosis [276]. Basal-like breast cancers are enriched for ferroptosis-associated polyunsaturated fatty acids (PUFAs), PUFA-containing phospholipids (PL-PUFA), and oxidized phospholipids (PL-PUFA-OOH), making them particularly sensitive to particularly sensitive to erastin and RSL3 [277]. In triple-negative breast cancer, higher plasma trimethylamine N-oxide (TMAO) levels associate with improved responses to immunotherapy [278], while the microbiota-derived metabolite phenylacetylglutamine (PAGln) suppresses T-cell activity and diminishes PD-1 antibody efficacy; fecal microbiota transplantation (FMT) can restore responsiveness in non-responders [279]. In colorectal cancer, enrichment of androsterone sulfate and dehydroepiandrosterone (DHEA) sulfate correlates with greater chemotherapy sensitivity [280]. Complementing these findings, metabolomics-based profiling of pancreatic ductal adenocarcinoma organoids delineates glucomet-PDAC (high glucose metabolism) and lipomet-PDAC (enhanced lipid metabolism); the glucomet subtype is more chemoresistant and portends worse prognosis, driven by a GLUT1-aldolase B (ALDOB)-glucose-6-phosphate dehydrogenase (G6PD) axis that reprograms glucose metabolism [281]. Taken together, directly targeting aberrant metabolites and classifying patients based on differences in metabolic characteristics, thereby guiding drug selection or determining therapeutic targets, provides a practical and feasible approach for personalized treatment.

## Challenges and future directions

Through non-invasive and patient-friendly sampling methods, metabolomics has identified numerous potential biomarkers for early cancer detection and prognosis for cancer. These biomarkers and abnormal metabolic enzymes have emerged as novel targets for therapeutic intervention. It is hoped that drug development targeting these targets will bring more effective treatments for patients. However, the clinical application of metabolomics remains limited. In this article, we review the main challenges faced by metabolomics in both technical and clinical aspects and propose metabolomics as a potentially effective detection approach for precise tumor treatment.

### Technical and clinical limitations

Although metabolomics shows great potential in oncology, the development and clinical application of metabolite-based therapies face several major challenges. These challenges, including both technical and clinical aspects, jointly hinder the transformation of metabolomics into personalized cancer treatments.

#### Technical limitations of metabolomics

Whether the sample collection and processing procedures are standardized largely determines the quality and consistency of metabolomics data. The differences in the patient’s fasting state, sample collection time and storage conditions will all affect the results of metabolomics, as well as the repeatability and reliability of metabolite measurement. Similarly, different analysis platforms and methods often produce different results, which makes it difficult to compare and integrate data from different studies. What is more serious is that the lack of standardized schemes for the extraction, detection and quantification of metabolites has further exacerbated this problem [282].

#### Clinical limitations of therapeutic targeting

One of the main challenges in developing metabolite-based targeted therapies is that they may produce off-target effects. Metabolites function through highly interconnected networks and participate in multiple biological processes simultaneously. Targeting a single metabolite or enzyme often leads to unexpected chain reactions. The similar structures of metabolic enzymes within the same family further exacerbate this challenge. Therefore, it is extremely difficult to design targeted drugs that can distinguish enzymes with similar structures. Generally, what is produced are small molecule inhibitors that affect multiple metabolic pathways. For instance, in a Phase I trial, the MAT2A inhibitor AG-270 demonstrated dose-limiting toxicity under high-dose exposure, including thrombocytopenia and acute liver injury. Mechanistically speaking, these adverse events may be related to the off-target inhibition of AG-270 on MAT1 (a structure-related enzyme crucial to liver function), ultimately leading to the decision to suspend its clinical development [283].

Another major challenge is acquired drug resistance. For instance, enasidenib has shown significant efficacy in AML patients with idh2 mutations, but some patients exhibit clinical resistance, disease progression, and elevated 2-HG levels [284]. Mechanistically, the acquired mutation of IDH prevents the formation of stable enzyme-inhibitor complexes and restores the production of 2-HG, thereby driving drug resistance in AML [285]. Similarly, the use of asparaginase can trigger a stress response, increase asparagine synthase (ASNS), and raise the intracellular asparagine level to meet the needs of tumors [286].

The patient’s metabolic profile was influenced by genetic background, lifestyle factors and comorbidities. This leads to clinical trials of metabolism-based treatments often showing heterogeneous responses among patients. For instance, studies on the glutaminase inhibitor CB-839 found that while some patients benefited, others did not, highlighting the necessity of improving patient stratification [252]. In addition, in patients with small cell lung cancer (SCLC), the efficacy of the MCT1 inhibitor AZD3965 is affected by the expression level of MCT4 [287].

### Emerging innovations by multi-omics

Unfortunately, due to the limitations of omics technology and the lack of unified and standardized sample collection methods, metabolomics data from cancer patients have not been well integrated. Therefore, promising metabolomics biomarkers have not yet been fully applied in clinical practice and are still mainly confined to the preclinical stage. In addition, the diversity of metabolomics analysis methods and the heterogeneity among patients may also hinder the clinical application of these research results. Therefore, for researchers, it is extremely difficult for us to identify metabolic markers with true functional relevance in noisy data.

A multi-omics approach that integrates genomic, transcriptomic, microbiome and metabolomic data clarifies the genetic and microbial origins of differential metabolites, providing additional therapeutic targets for cancer treatment. For instance, through a comprehensive analysis of genomic, transcriptomic and metabolomics data, TNBC was classified into three distinct subtypes, and the key metabolites and potential therapeutic targets specific to each subtype were identified [21].

Another study, by jointly analyzing metabolomics and genomic data, depicted different subgroups in the NPM1-mutated AML cohort. Research has found that AML patients with NPM1/polymerin mutations have stronger NAD and purine metabolism disorders, which has identified potential therapeutic targets for the treatment of this subgroup of patients [6].

### Preventive and lifestyle interventions

In addition to the imbalance of metabolites caused by abnormal metabolic enzymes, unhealthy diet and lifestyle can also promote the increase of carcinogenic metabolites. Among them, the imbalance of intestinal flora is particularly worthy of attention. For instance, acetaldehyde, the main metabolite of ethanol, can cause pathological changes in the gastrointestinal tract, liver, pancreas and gallbladder. Among drinkers, the risk of colorectal cancer and other cancers increases significantly [288]. Unhealthy dietary patterns, sedentary behaviors and obesity are the main factors leading to the occurrence of colorectal cancer [289]. On the contrary, regular exercise has been proven to stimulate microbial single-carbon metabolism, increase formic acid levels, enhance the function of cytotoxic CD8 T cells, and improve the efficacy of immune checkpoint inhibitors [290]. Therefore, a healthy intestinal flora not only maintains the balance within the intestines but also has anti-cancer effects. These results demonstrate the significance of a healthy lifestyle in preventing and reducing the risk of cancer.

### Promising metabolites and therapeutic targets

Some metabolites and metabolic enzymes have become particularly promising targets in cancer treatment. In carbohydrate metabolism, tumor metabolites such as 2-HG, succinate, fumarate and lactic acid are effective targets for treating tumors. The approval of IDH inhibitors such as ivosidenib and the development of MCT1 inhibitors such as AZD3965 prove this point. There are also many targets in amino acid metabolism, including identified targets such as asparagine, glutaminase, and the rate-limiting enzyme PHGDH for serine synthesis, as well as targets like arginase 1 and IDO1 that regulate the immune microenvironment. In nucleotide metabolism, inhibiting ribonucleotide reductase remains the basis of chemotherapy. The new strategy focuses on regulating the upstream/downstream, such as treating MTAP-deficient tumor cells with L-alanine and antagonizing the A2A receptor or inhibiting CD73 to counteract extracellular adenosine signaling. In addition, targeting lipid metabolism through fatty acid synthase (such as TVB-2640) and regulating the intestinal microbiota through fecal microbiota transplantation are highly promising therapeutic approaches. These abundant targets indicate that targeted metabolism can effectively inhibit tumor growth, overcome immunosuppression and improve cancer treatment.

In conclusion, despite substantial progress made in identifying metabolomics biomarkers and therapeutic targets, clinical applications are still affected by the diversity of metabolomics platforms and the lack of unified standards. In addition, issues such as off-target effects, acquired drug resistance, and patient heterogeneity make the development of metabolite-based therapies difficult. In order for metabolomics to be truly applied in clinical treatment, the standardization of full-process metabolomics needs to be established. To achieve precise stratification and individualized medication for patients, the combined analysis of multi-omics and clinical phenotypes is necessary.

## Data Availability

Not applicable.
